# The Relationship between High Risk for Obstructive Sleep Apnea and General and Central Obesity: Findings from a Sample of Chilean College Students

**DOI:** 10.1155/2014/871681

**Published:** 2014-04-14

**Authors:** Adaeze C. Wosu, Juan Carlos Vélez, Clarita Barbosa, Asterio Andrade, Megan Frye, Xiaoli Chen, Bizu Gelaye, Michelle A. Williams

**Affiliations:** ^1^Department of Epidemiology, Multidisciplinary International Research Training Program, Harvard School of Public Health, 677 Huntington Avenue, Kresge 500, Boston, MA 02115, USA; ^2^Centro de Rehabilitación Club de Leones Cruz del Sur, Suiza 1441, Punta Arenas, 6211525 Magallanes, Chile

## Abstract

This cross-sectional study evaluates the prevalence and extent to which high risk for obstructive sleep apnea (OSA) is associated with general obesity and central obesity among college students in Punta Arenas, Chile. Risk for OSA was assessed using the Berlin Questionnaire and trained research nurses measured anthropometric indices. Overweight was defined as body mass index (BMI) of 25–29.9 kg/m^2^ and general obesity was defined as BMI ≥ 30 kg/m^2^. Central obesity was defined as waist circumference ≥90 centimeters (cm) for males and ≥80 cm for females. Multivariate logistic regression models were fit to obtain adjusted odds ratios (OR) and 95% confidence intervals (CI). Prevalence of high risk for OSA, general obesity, and central obesity were 7.8%, 12.8%, and 42.7%, respectively. Students at high risk for OSA had greater odds of general obesity (OR 9.96; 95% CI: 4.42–22.45) and central obesity (OR 2.78; 95% CI 1.43–5.40). Findings support a strong positive association of high risk for OSA with obesity.

## 1. Background

Chile, an upper middle-income country in South America, has seen increases in the prevalence of overweight and obesity, well-established risk factors for conditions such as cancer, cardiovascular disease, and diabetes [1, 2]. In 1987, the prevalence of excess weight among 6-year-olds in Chile was 13%; by 2000, it had reached 27% [3]. According to a 2012 report by the Organization for Economic Cooperation and Development (OECD), 25.1% of Chilean adults are obese and, among Chilean children aged 5–17 years, 27.1% of girls and 28.6% of boys are overweight or obese [4]. Like excess weight, sleep disorders, particularly sleep apneas, have been associated with hypertension [5, 6], coronary artery disease [7], myocardial infarction [8, 9], heart failure [10], and diabetes [9]. Although reports have been published on the prevalence of obesity and of sleep apnea in Chilean adults and young children [4, 11], few reports have focused attention on the prevalence of obesity and sleep apnea among young adults, particularly college students transitioning from adolescence into adulthood. Moreover, the college years are often characterized by insufficient sleep, increased consumption of energy drinks and caffeinated beverages, and irregular meal and sleep patterns, which can have important consequences for overall health and wellbeing [12–15]. To address the paucity of literature on the epidemiology of obstructive sleep apnea (OSA) and adiposity indices among college students in Chile, and to lay groundwork for developing health promotion and disease prevention strategies, we conducted a cross-sectional study of students at four Chilean colleges. We investigated OSA risk and the extent to which high risk for OSA was associated with overweight, general obesity, and central obesity in this population.

## 2. Methods

### 2.1. Study Population

The study was conducted between December 2010 and June 2011 at four colleges in the Magallanes region of Chile: Universidad Tecnologica de Chile, Universidad del Mar, Universidad de Magallanes, and Universidad Santo Tomas. Detailed descriptions of the study procedures have been given previously [16]. Of the 994 full-time undergraduates who participated in the study, we excluded students who were above 35 years old or missing information on age, sleep apnea (i.e., The Berlin Questionnaire), or adiposity measures (*n* = 78). The final sample consisted of 916 students aged 18 to 35 who had complete exposure and outcome information.

### 2.2. Recruitment

Flyers promoting the study were posted throughout departments at the participating colleges. Interested students were invited to meet in a large room or an auditorium where they were informed about the study purpose and procedures and invited to complete anonymous questionnaires requesting demographic, lifestyle, and sleep-related information. Students who gave informed consent and completed the questionnaires were invited to participate in a brief physical examination during which trained research nurses took cardiometabolic and anthropometric measures including height, weight, blood pressure (BP), pulse rate, waist circumference (WC), and hip circumference (HC). All study procedures were approved by the institutional review boards of Centro de Rehabilitación Club de Leones Cruz del Sur, Punta Arenas, Chile, and the University of Washington, USA. The Harvard School of Public Health Office of Human Research Administration, USA, granted approval to use the anonymized dataset for analyses.

### 2.3. Obstructive Sleep Apnea (OSA) Risk

OSA risk was evaluated using the Berlin Questionnaire. Since it was first developed in 1996, the Berlin Questionnaire has been validated and used extensively in clinical and epidemiologic research globally including Latin America [17–23]. Numerous validation studies have reported moderate to high consistency between results of the Berlin Questionnaire and parameters of polysomnography, particularly respiratory disturbance index (RDI) > 5 and apnea-hypopnea index (AHI) ≥ 5, especially within clinical populations [24].

The questionnaire is divided into three categories [19, 25]. Category 1 evaluates snoring characteristics and how often the respondent ceases breathing during sleep. Category 2 assesses frequency of feelings of fatigue or tiredness after waking as well as sleepiness while driving. Category 3 ascertains information about obesity and hypertension [17, 19]. In the first two categories, a participant is given a positive score if he or she responds with “yes” or experiences frequent symptoms (more than 3 to 4 times per week) in two or more questions within that category. For category 3, a participant is given a positive score if he or she is obese (BMI ≥ 30 kg/m^2^) or has a history of hypertension. Participants with positive scores in two or more categories are classified as having a high risk for OSA [17].

### 2.4. General Obesity

Body mass index (BMI) was calculated as weight in kilograms divided by height in square meters (kg/m^2^). Categories of BMI were defined according to the World Health Organization (WHO) criteria (underweight: <18.5 kg/m^2^; normal: 18.5–24.9 kg/m^2^; overweight: 25.0–29.9 kg/m^2^; and obese: ≥30.0 kg/m^2^). General obesity was defined as BMI ≥ 30.0 kg/m^2^ [26].

### 2.5. Central Obesity

Central obesity was evaluated using the International Diabetes Federation (IDF) criteria for South American men and women, that is, WC ≥ 90 cm (for men) and WC ≥ 80 cm (for women) [27]. Waist-to-hip ratio (WHR) was calculated by dividing WC by HC. WHR was further categorized by quartile distribution and central obesity defined as WHR in the top quartile. Each anthropometric measurement was taken twice, and the average was used in data analyses.

### 2.6. Covariates

Sociodemographic information collected included age, sex, and year in college. Lifestyle and behavioral characteristics included moderate or vigorous physical activity (yes versus no), cigarette smoking status (never, former, or current), alcohol consumption within the past year (no versus yes), and use of more than one energy or stimulant drink per week (no versus yes). Energy or stimulant drinks were defined as beverages used to boost energy levels, increase alertness, and enhance cognition and mood [16]. Examples of these beverages include coffee, yerba mate, cola, Red Bull, and Dark Dog. Other variables included self-rated health (good versus poor) and systolic and diastolic BP.

### 2.7. Statistical Analysis

High risk for obstructive sleep apnea (OSA), defined as described above, was the exposure and the outcomes of interest were overweight, general obesity, central obesity, BMI, WC, HC, and WHR. First, we examined the frequency distributions of sociodemographic, lifestyle, and behavioral characteristics of the students. Characteristics of participants were summarized using counts and percentages for categorical variables and mean values (± standard deviations) for continuous variables. Student's* t*-tests and chi-square tests were used to calculate bivariate differences for continuous and categorical variables, respectively. We examined the frequency distribution of OSA risk according to sociodemographic, lifestyle, and behavioral characteristics. Multivariate linear regression models were used to assess the associations of high OSA risk with BMI, WC, HC, and WHR. Multivariate logistic regression models were used to determine odds ratios (OR) and 95% confidence intervals (CI) of the association of high OSA risk with general obesity (BMI ≥ 30 kg/m^2^), central obesity according to the IDF criteria, and WHR in the top quartile. Confounding variables were considered* a priori* on the basis of their hypothesized relationship with OSA and adiposity. Potential confounders included age, sex, year in college, alcohol consumption, use of energy drinks, cigarette smoking status, self-rated health, BP, and participation in moderate or vigorous physical activity. We conducted stratified analyses to evaluate the extent to which, if at all, associations of high OSA risk with overweight, general obesity, and central obesity differed by sex, elevated BP, and subjects' self-rated health. All statistical analyses were performed using Statistical Analysis Software (SAS, version 9.3; SAS Institute, Cary, NC). All *P* values are two-sided and set at *α* = 0.05.

## 3. Results

**Figure 1:**
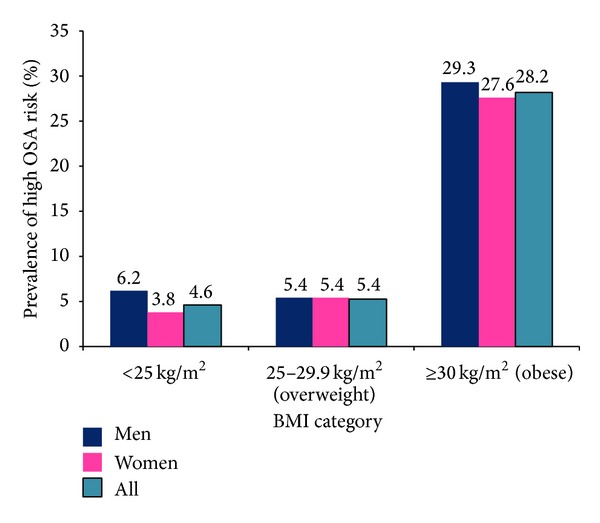
Prevalence of high OSA risk according to BMI category and gender.

**Figure 2:**
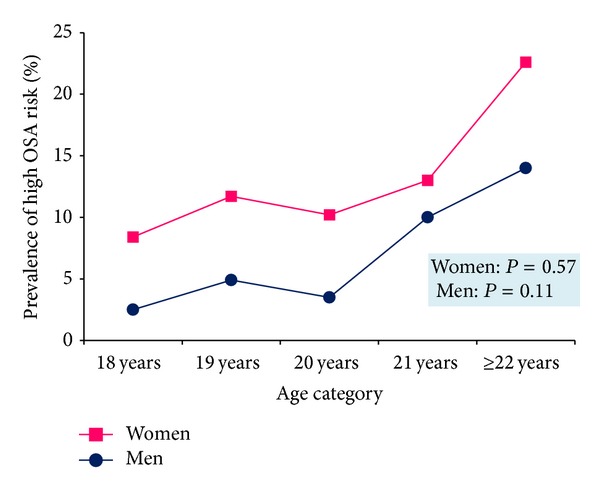
Prevalence of high OSA risk according to age group and gender.

Of the 916 students included in the analysis, 69.5% were females and the average reported age was 21.8 (±3.3) years. Approximately 7.8% were classified as having high risk for OSA as determined using previously published scoring and classification scheme of the Berlin Questionnaire. Approximately 28.3% of students were overweight (BMI: 25–29.9 kg/m^2^) and 12.8% had general obesity according to WHO criteria (BMI ≥ 30 kg/m^2^). Using the IDF definition, it was determined that 42.7% of students had central obesity (WC ≥ 90 cm for men or WC ≥ 80 cm for women). High risk for OSA was associated with age, poor self-rated health, and cigarette smoking status. However, high risk for OSA was not associated with sex, year in college, systolic BP, diastolic BP, alcohol consumption, participation in moderate to vigorous physical activity, or consumption of more than one energy or stimulant drink per week (Table 1). Prevalence of high OSA risk was highest in the obese BMI category (Figure 1) and in individuals who were over 21 years old (Figure 2).

Table 2 shows the results of the fully adjusted models of the linear regression analyses. High OSA risk was statistically significantly associated with higher BMI (*β*: 3.25; standard error: 0.63, *P* < 0.001) and WHR (*β*: 0.03; standard error: 0.01, *P* = 0.02). High OSA risk was also associated with higher WC (*β*: 7.32; standard error: 1.54, *P* < 0.001) and HC (*β*: 5.00; standard error: 1.41, *P* < 0.05).

Next, we completed logistic regression analyses. In the fully adjusted models, high risk for OSA was associated with almost 10-fold increased odds of general obesity (OR 9.96; 95% CI 4.42–22.45) compared to low risk for OSA. Students with high OSA risk also had 2.78-fold odds of central obesity (OR 2.78; 95% CI 1.43–5.40). However, the odds of high risk for OSA were not elevated by overweight status (OR 0.89; 95% CI 0.35–2.23). Those with high risk for OSA were also more likely to be in the top quartile of WHR (i.e., WHR ≥ 0.8875), although statistical significance was not achieved (OR 1.45; 95% CI 0.71–2.95) (Table 3).

Similar strong positive associations between high risk for OSA and general obesity (BMI ≥ 30 kg/m^2^) were evident within strata of sex (male or female), BP (nonelevated or elevated), and self-rated health (good or poor). High risk for OSA was not associated with overweight in strata of sex, BP, or self-rated health. For central obesity as defined by the IDF, the results of the stratified analyses were mixed. High risk for OSA was associated with central obesity only in men (OR 4.66; 95% CI: 1.33–16.39), in individuals with elevated BP (OR 7.34; 95% CI: 2.38–22.59), and in those with good self-rated health (OR 2.60; 95% CI 1.28–5.28) while the findings in women and those with poor self-rated health were suggestive of a positive though not statistically significant association (Table 4).

## 4. Discussion

In this study of Chilean college students, 7.8% were classified as having high risk for OSA as defined by the Berlin Questionnaire and there was a high prevalence of overweight (28.3%), general obesity (12.8%), and central obesity (42.7%). High OSA risk was strongly and positively associated with general obesity and central obesity, but not with overweight.

### 4.1. Possible Biological Mechanisms Explaining the Relationship between OSA and Obesity

Although the exact pathway linking OSA and obesity remains somewhat unclear, it is recognized that obesity contributes to airway collapse during sleep by causing increased fat deposits around the upper airway, thus narrowing it and diminishing the activity of the muscles in that region [28–31]. Conversely, sleep apnea may contribute to excess weight and obesity through sleep loss and daytime sleepiness as apneic individuals rouse often to resume breathing and reenable unobstructed air flow [29]. Moreover, insufficient sleep has been associated with increased energy need and food intake [32]. On balance, available evidence suggests mechanisms that may underlay bidirectional relationships between increased adiposity and OSA.

### 4.2. Association between OSA and Obesity

We found strong positive associations of high OSA risk with general obesity, central obesity, BMI, and WHR. These findings are largely consistent with findings from numerous prior studies [33–40]. In the Wisconsin Sleep Cohort Study, a 10% weight gain was associated with approximately 32% increase in AHI as assessed by polysomnography [38]. In their study of patients at primary health care centers in Dubai, Mahboub et al. found that approximately 70% of those at high risk for OSA had a BMI ≥ 30 kg/m^2^ while about 75% of the low risk group had BMI < 30 kg/m^2^ [41]. Likewise, Blondet et al. found in a study of middle-aged Puerto Ricans that among men with BMI < 30 kg/m^2^, 98% had high risk for OSA compared to 50% among nonobese men. In addition, the authors noted that 70% of women with BMI > 28 kg/m^2^ were at high risk for OSA compared to only 28% of those with BMI < 28 kg/m^2^ [42]. The prevalence of high risk for OSA among students with general obesity in our study was substantially higher (28.3%) compared to that in normal weight (4.6%) and overweight (5.4%) students. Although participants in many studies examining obesity and OSA risk are older and heavier than our population, significant positive associations between sleep apnea and obesity have also been found in pediatric and adolescent populations [43–46].

While we found no association between overweight BMI category and high risk for OSA in our sample, Peppard et al. observed a positive association between overweight BMI category and AHI in the Wisconsin Sleep Cohort Study [47]. Despite our finding of no association, overweight students should be targeted for interventions as these students are at higher risk for obesity in the near future compared to normal weight students.

### 4.3. Prevalence of High Risk for OSA

The prevalence of high risk for OSA in our sample (7.8%) is higher than what is reported among US young adults. For instance, authors of a study among 1,845 students enrolled in a southeastern US college reported a prevalence of 4% as assessed using the SLEEP-50 survey [48]. However, our finding is lower than high OSA risk estimates reported for US adults (26%) who participated in the 2005 Sleep in America Poll [40]. We found no significant difference in frequency of OSA risk by sex (9.3% for men and 7.1% for women; *P* = 0.25) within our sample, although other investigators have shown that men have a higher risk for OSA compared to women—the main hypothesis being that men tend to have more fat deposits in the upper part of the body, that is, around the neck, trunk, and abdomen [49].

### 4.4. Prevalence of Obesity

The prevalence of general obesity in our study (12.8%) was relatively low when compared to the prevalence reported for Chilean adults by the OECD (25.1%) or that determined from a multistage cluster sampling of adults ≥40 years old in Santiago, Chile (31.7%) [4, 11]. However, the combined prevalence of overweight and obesity (i.e., BMI ≥ 25 kg/m^2^) in our study was greater than that observed in a study of 3,461 Chilean students aged 17–24 years (41.1% versus 19%) [50]. When we examined the frequency of BMI ≥ 25 kg/m^2^ by age within our sample, there was still a high prevalence of excess weight in the respective groups: 18–24 year-olds (39.5%) and 25–35 year-olds (48.4%); *P* = 0.04. The overall prevalence of central obesity as defined using the IDF criteria was 42.7%. These high frequencies of overweight and obesity in young adulthood are of concern as they may have implications for the development of cardiometabolic conditions in later life.

### 4.5. Strengths and Limitations

Our study has several strengths. First, numerous validation studies have reported moderate to high consistency between results of the Berlin Questionnaire and parameters of polysomnography, the gold-standard for diagnosing sleep disorders [24]. Second, we employed various statistical methods and assessed the relationship between high OSA risk and various indices of adiposity. Third, the likelihood for misclassification of obesity was reduced as trained research nurses used standard protocols to take anthropometric measurements, thus avoiding reliance on students' self-report.

There are some limitations that should be considered when interpreting the results of our study. Given our cross-sectional study design, we are unable to determine if high risk for OSA preceded obesity. Longitudinal studies are needed to clarify the temporal relationship between OSA and obesity. Another limitation is that participants were not sampled randomly; rather, we relied on subjects' willingness to participate. As such, our findings could be subject to volunteer bias. Approximately 8% of students were excluded because of incomplete information on sleep apnea or anthropometric data. We found no significant difference between the prevalence of poor self-rated heath among individuals in our sample and those who were excluded (12.1% versus 6.8%; *P* = 0.29) nor any significant difference in the distribution of cigarette smoking status (*P* = 0.28). However, excluded students tended to be older than those in our sample (mean ± SD age: 23.4 ± 5.0 versus 21.8 ± 3.3; *P* < 0.01). Given that OSA risk increases with age, it is possible that we missed individuals with high OSA risk among those who had incomplete sleep apnea or anthropometric information. Thus, the prevalence of high risk for OSA in our study could be an underestimate. Conversely, given the high prevalence of obesity within this sample, the use of odds ratios may give an exaggeration of the true estimate. We cannot exclude the possibility of residual confounding by unmeasured factors such as socioeconomic status, dietary intake (e.g., consumption of fruits, vegetables, or fast foods), or student's course load. Finally, due to the relatively small sample size, the 95% confidence intervals for the estimates from the logistic regression and stratified analyses are quite large and imprecise.

## 5. Conclusion

Our findings support significant positive associations of high OSA risk with general obesity and central obesity. Additional longitudinal evidence is needed to establish the temporal relationship of OSA and obesity. If our findings are confirmed, health care services for college students should include health promotion and disease prevention strategies about the importance of maintaining healthy weight. Strategies should also include health education modules that may be used to improve students' knowledge about sleep disorders, as prevention and control of cardiometabolic risk factors in young adulthood may have implications for preventing and mitigating chronic disease burden in later life.

## Figures and Tables

**Table 1:** Characteristics of 916 college students in Chile, according to OSA risk.

Characteristic	Overall (*n* = 916)	OSA risk		*P* value^c^
		Low (*n* = 845)	High (*n* = 71)	
*Demographic characteristics *				
Age, year, mean (SD)	21.8 (3.3)	21.7 (3.3)	22.5 (3.4)	0.0425
Male, %	30.5	30.0	36.6	0.2429
Education (year in college), %				
First year	37.3	37.4	35.7	0.4826
Second year	30.7	30.5	34.3	
Third year	18.5	19.0	12.9	
Fourth+ year	13.4	13.1	17.4	
*Lifestyle factors *				
Any physical activity participation, %	53.9	55.5	52.1	0.5811
Alcohol consumption (past year), %	80.3	80.0	85.1	0.3105
Smoking status, %				
Nonsmoker	42.7	44.2	24.6	0.0051
Current	42.4	40.9	60.0	
Former	14.9	14.9	15.4	
Use of >1 energy drinks per week, %	54.3	53.8	60.6	0.2866
*Self-rated health and blood pressure *				
Self-rated health^a^ (poor), %	12.1	11.4	22.2	0.0183
Systolic blood pressure (mmHg)	121.5 (14.4)	121.3 (14.3)	124.6 (15.6)	0.0642
Diastolic blood pressure (mmHg)	74.2 (11.7)	74.0 (11.8)	76.5 (10.8)	0.0932
*Anthropometric measurements *				
BMI, kg/m^2^, mean (SD)	24.9 (4.5)	24.6 (4.2)	28.6 (6.0)	<0.0001
WHO criteria of overweight and obesity (kg/m^2^), %				
Underweight (BMI <18.5)	1.5	1.7	0.0	<0.0001
Normal weight (18.51–24.9)	57.4	59.4	33.8	
Overweight (BMI: 25–29)	28.3	29.0	19.7	
Obese (BMI ≥30)	12.8	9.9	46.5	
Central obesity^b^, %	42.7	40.5	69.0	<0.0001
Waist circumference, cm, mean (SD)	81.9 (11.9)	81.1 (11.4)	91.0 (13.9)	<0.0001
Hip circumference, cm, mean (SD)	97.9 (10.0)	97.3 (9.7)	104.6 (11.0)	<0.0001
Waist-to-hip ratio, mean (SD)	0.84 (0.08)	0.83 (0.08)	0.87 (0.9)	0.0007
Waist-to-hip ratio, top quartile (WHR ≥ 0.8875), %	24.9	23.8	36.6	0.0162

SD: standard deviation; PA: physical activity; BP: blood pressure; BMI: body mass index.

^a^Self-rated health (SRH) was evaluated by asking, “how would you say your health compares to others your age.” Poor SRH = worse than others my age. Good SRH = better or same as others my age.

^b^Based on the International Diabetes Federation (IDF) criteria for the definition of central obesity among South Americans: waist circumference ≥90 cm for men; waist circumference ≥80 cm for women.

^c^Student *t*-test for continuous variables; chi-square for categorical variables.

**Table 2:** Linear regression analyses: associations of high OSA risk with anthropometric measurements among 916 college students in Chile.

Model	BMI (kg/m^2^)		Waist-to-hip ratio (cm)		Waist circumference (cm)		Hip circumference (cm)	
	*β* (SE)	*P* value	*β* (SE)	*P* value	*β* (SE)	*P* value	*β* (SE)	*P* value
Model 1: unadjusted	3.93 (0.54)	<0.0001	0.034 (0.01)	0.0007	9.84 (1.43)	<0.0001	7.30 (1.21)	<0.0001
Model 2: adjusted for demographic factors^a^	3.76 (0.54)	<0.0001	0.027 (0.01)	0.0037	8.94 (1.37)	<0.0001	7.03 (1.21)	<0.0001
Model 3: adjusted for demographic & lifestyle factors^b^	4.20 (0.58)	<0.0001	0.028 (0.01)	0.0061	9.25 (1.47)	<0.0001	7.37 (1.30)	<0.0001
Model 4: Model 3 + blood pressure^c^	3.97 (0.55)	<0.0001	0.026 (0.01)	0.0101	8.59 (1.38)	<0.0001	6.83 (1.24)	<0.0001
Model 5: Model 4 + self-rated health^d^	3.25 (0.63)	<0.0001	0.028 (0.01)	0.0193	7.32 (1.54)	<0.0001	5.00 (1.41)	0.0004

^a^Demographic factors included age (continuous), sex, and education level.

^b^Lifestyle factors included any physical activity participation, alcohol consumption (within the past year—yes/no), cigarette smoking (never, former, or current), and use of energy drinks (yes/no).

^c^Continuous blood pressure variables.

^d^Self-rated health (SRH) was evaluated by asking, “how would you say your health compares to others your age.” Poor SRH = worse than others my age. Good SRH = better or same as others my age.

**Table 3:** Logistic regression analyses: associations of high OSA risk with overweight and obesity among 916 college students in Chile.

Model	WHO criteria	WHO criteria		IDF criteria^f^
	BMI: 25–29 versus BMI <25	BMI ≥30 versus BMI <25	WHR ≥0.8875 versus WHR <0.8875^e^	Central obesity versus no central obesity
	OR (95% CI)	OR (95% CI)	OR (95% CI)	OR (95% CI)
Model 1: unadjusted	1.23 (0.63, 2.42)	8.45 (4.76, 15.00)	1.85 (1.11, 3.08)	3.27 (1.94, 5.51)
Model 2: adjusted for demographic factors^a^	1.09 (0.55, 2.17)	8.26 (4.59, 14.86)	1.65 (0.96, 2.86)	3.26 (1.92, 5.56)
Model 3: adjusted for demographic and lifestyle factors^b^	1.53 (0.74, 3.17)	10.24 (5.30, 19.81)	1.68 (0.93, 3.05)	3.37 (1.90, 5.97)
Model 4: Model 3 + blood pressure^c^	1.59 (0.76, 3.36)	10.92 (5.39, 22.11)	1.59 (0.87, 2.91)	3.31 (1.85, 5.95)
Model 5: Model 4 + self-rated health^d^	0.89 (0.35, 2.23)	9.96 (4.42, 22.45)	1.45 (0.71, 2.95)	2.78 (1.43, 5.40)

BMI: body mass index; WHR: waist-to-hip ratio; IDF: International Diabetes Federation; WC: waist circumference; OR: odds ratio; 95% CI: 95% confidence interval.

^a^Demographic factors included age (continuous), sex, and education level.

^b^Lifestyle factors included any physical activity participation, alcohol consumption (within the past year—yes/no), cigarette smoking (never, former, or current), and use of energy drinks (yes/no).

^c^Continuous blood pressure variables.

^d^Self-rated health (SRH) was evaluated by asking, “how would you say your health compares to others your age.” Poor SRH = worse than others my age. Good SRH = better or same as others my age.

^e^WHR ≥0.8875 refers to the top quartile of WHR.

^f^Central obesity as defined by the International Diabetes Federation (IDF): WC ≥90 cm for men and WC ≥80 cm for women in South America.

**Table 4:** Stratified analysis for the association of high OSA risk with overweight, general obesity, and central obesity, by sex, blood pressure, and self-rated health^1^.

Stratified variable	All participants *N* = 916	WHO criteria BMI: 25–29 versus <25 OR (95% CI)^a^	WHO criteria BMI ≥30 versus <25 OR (95% CI)^a^	IDF criteria^b^ Central obesity versus no central obesity
Sex				
Male	280	0.56 (0.13, 2.47)	7.23 (1.34, 38.94)	4.66 (1.33, 16.39)
Female	636	1.16 (0.34, 3.95)	14.96 (5.40, 2.30)	2.08 (0.90, 4.81)
Elevated blood pressure^c^				
No	381	0.58 (0.12, 2.94)	8.71 (2.26, 33.59)	1.12 (0.39, 3.22)
Yes	534	1.21 (0.36, 4.09)	12.42 (4.23, 36.48)	7.34 (2.38, 22.59)
Self-rated health^d^				
Good	667	0.72 (0.25, 2.07)	8.21 (3.52, 19.16)	2.60 (1.28, 5.28)
Poor	92	3.01 (0.16, 57.86)	144.82 (2.4, ∞)	15.76 (0.93, 267.69)

BMI: body mass index; IDF: International Diabetes Federation; OR: odds ratio; 95% CI: 95% confidence interval.

^a^Except for the stratified variables, the following variables were adjusted for age (continuous), sex, education, any physical activity participation, alcohol consumption, cigarette smoking, use of energy drinks, and self-rated health.

^b^Central obesity was defined by the International Diabetes Federation (IDF) criteria for South Americans: WC ≥90 cm for men and WC ≥80 cm for women.

^c^Elevated BP defined as systolic BP ≥120 mmHg or diastolic BP ≥80 mmHg.

^d^Self-rated health (SRH) was evaluated by asking, “how would you say your health compares to others your age.” Poor SRH = worse than others my age. Good SRH = better or same as others my age.
